# Motif-Based Text Mining of Microbial Metagenome Redundancy Profiling Data for Disease Classification

**DOI:** 10.1155/2016/6598307

**Published:** 2016-02-14

**Authors:** Yin Wang, Rudong Li, Yuhua Zhou, Zongxin Ling, Xiaokui Guo, Lu Xie, Lei Liu

**Affiliations:** ^1^Shanghai Public Health Clinical Center and Institutes of Biomedical Sciences, Fudan University, Shanghai 200032, China; ^2^Shanghai Center for Bioinformation Technology, Shanghai 201203, China; ^3^Key Lab of Computational Biology, CAS-MPG Partner Institute for Computational Biology, Shanghai Institutes for Biological Sciences, Chinese Academy of Sciences, Shanghai 200031, China; ^4^Department of Medical Microbiology and Parasitology, Institutes of Medical Sciences, Shanghai Jiao Tong University School of Medicine, Shanghai 200240, China; ^5^Collaborative Innovation Center for Diagnosis and Treatment of Infectious Diseases, State Key Laboratory for Diagnosis and Treatment of Infectious Diseases, The First Affiliated Hospital, School of Medicine, Zhejiang University, Hangzhou, Zhejiang 310003, China

## Abstract

*Background*. Text data of 16S rRNA are informative for classifications of microbiota-associated diseases. However, the raw text data need to be systematically processed so that features for classification can be defined/extracted; moreover, the high-dimension feature spaces generated by the text data also pose an additional difficulty.* Results*. Here we present a Phylogenetic Tree-Based Motif Finding algorithm (PMF) to analyze 16S rRNA text data. By integrating phylogenetic rules and other statistical indexes for classification, we can effectively reduce the dimension of the large feature spaces generated by the text datasets. Using the retrieved motifs in combination with common classification methods, we can discriminate different samples of both pneumonia and dental caries better than other existing methods.* Conclusions*. We extend the phylogenetic approaches to perform supervised learning on microbiota text data to discriminate the pathological states for pneumonia and dental caries. The results have shown that PMF may enhance the efficiency and reliability in analyzing high-dimension text data.

## 1. Introduction

The microbial ecology in human determines or promotes necessary bioprocesses in human bodies, and compositions of microbial communities can be reflections for the health conditions of the hosts [1]. In fact, the complex microbial communities play key roles in human health from time to time. For example, dysfunction of microbiota biogeography or infection of pathogenic microbiota would lead to a series of human diseases, like pneumonia [2], dentes cariosus [3], and so on [4, 5]. Fortunately, sequencing of 16S rRNA provides informative knowledge for the distributions of microbiota [6]. For instance, microbiota taxonomy analysis based on the sequence data by bioinformatic tools such as Ribosomal Database Project (RDP) website would facilitate investigations of key microorganisms associated with certain host diseases [7].

On the other hand, traditional (supervised) methods such as feature selection are frequently adopted in classifications of microbiota-associated disease samples, for example, selecting the microorganism(s) which can maximally discriminate diseased and healthy hosts [6]. Nonetheless, substantial amounts of sequence data actually embody the characteristics of entire microbial communities rather than individual microbes [8]. Hence, the mapping results of 16S rRNA segments to individual microbes based on the sequencing data would not be informative enough for the aftermath feature selection. Furthermore, sequences that cannot be mapped to known microbes might also have certain importance. Therefore, algorithms focusing on the textual features of microbiota sequences themselves (e.g., *k*-mer/*k*-tun features) have been applauded by recent researchers, as they skip the sequence-microbe mapping and hence avoid the intrinsic drawbacks [9, 10].

However, abundantly many features can be defined regarding raw text data (i.e., strings); in other words, the dimension of feature space would usually be extremely high; thus the “curse of dimensionality” resulted [11]. In this regard, motif-oriented algorithms are capable of accelerating the feature selection pipeline, as generalizing or lumping the textual features of a lot of strings into certain motifs is equivalent to degenerating the feature space (i.e., dimension reduction) [12, 13]. Nonetheless, extracting the motifs put forward another issue, intuitively because motifs can be defined in various different ways and there is no universal solution. Therefore, systematic approaches for motif extraction/definition are necessary.

For this purpose, here we present an improved text mining method named Phylogenetic Tree-Based Motif Finding algorithm (PMF). In this method, relevance between text strings is considered, which are defined by the phylogeny of the strings. By statistically associating the motif counts computed via PMF with disease statuses, efficient classification of disease samples based on (microbiota) sequence texts could be achieved. We have simulated the 16S rRNA datasets of pneumonia and dentes cariosus patients with this pipeline, respectively. Compared to previous results [14], our new pipeline shows better classifications. Additionally, the pipeline is suitable for issues with high-dimensional feature spaces.

## 2. Data and Methods

### 2.1. Data and Preprocessing

We acquired 16S rRNA sequencing fasta files of pneumonia patients and dental decay patients from Zhou et al. [2] and Ling et al. [3], respectively. Two to six length *k*-mer counting results in each meta-genomic sequence were calculated [15]. The *k*-mer counting results were shown in Files S1 and S2 in Supplementary Material available online at http://dx.doi.org/10.1155/2016/6598307. Each counting and its antisense complementary result were summarized and combined together. The *k*-mer frequencies were normalized by the reciprocal of length of each sequence as weight and divided by the number of sequences in each fasta file. Identified microbes of 16S rRNAs' sequences from Zhou et al. [2], which were downloaded from NCBI website (ID: GU737566 to GU737625 and HQ914698 to HQ914775) (http://www.ncbi.nlm.nih.gov), were used for constructing the phylogenetic trees. After removing redundant sequences, a total of 90 microbe species were used for further analysis.

The pneumonia samples included 101 patients with hospital-acquired pneumonia (HAP), 43 patients with community-acquired pneumonia (CAP), and 42 normal persons as control. 71 HAP cases, 32 CAP cases, and 30 cases of normal samples were allocated as training data; fitness was calculated using 5-fold proportional cross validation. The other 30 cases of HAP, 13 cases of CAP, and 12 cases of normal were set as the test data, so that classifications were evaluated. For the *k*-mer counting profiles of 16S rRNAs fasta file collected from dental plaques samples, the training data contained 23 dental decay patients and 20 normal samples and the test data contained 9 dental decay patients and 8 normal samples. For the *k*-mer counting of 16S rRNAs collected from saliva samples, the training data contained 23 dental decay patient samples and 19 normal samples; and the test data contained 10 dental decay patient samples and 8 normal samples. The partition of the training and test datasets, as well as the cross validation of training data themselves, was adopted from the previous study; hence impartial comparisons (with previous results [14]) could be performed.

### 2.2. Phylogenetic Tree-Based Motif Finding (PMF) Method

The improved text mining method, Phylogenetic Tree-Based Motif Finding algorithm (PMF), handled counting results of each person's 16S rRNA fasta file. PMF algorithm consisted of three parts: motif finding, motif sorting, and model evaluation. Motif finding was the main part of the algorithm, in which key step was constructing a clustering tree to combine the original strings to a new motif, that is, transforming original letters (“A,” “T,” “C,” and “G”) into the generalized letters (“Y,” “R,” “W,” “K,” “M,” “S,” “D,” “V,” “B,” “H,” and “N”). The rules of the generalized letters were shown in Table 1.

Minimum distance method was used to cluster the phylogenetic tree. For each pair of sequences with the same length, the phylogenetic distance was calculated by summarizing differences of all sites. For the generalized letters, the differences were calculated using the number of intersections divided by the number of unions. The phylogenetic distance of two motifs was estimated by summarizing differences of both original and generalized sites. If the phylogenetic distance of antisense complementary sequence was smaller than the original sequence, the instance of its antisense complementary sequence was selected. To calculate the complementary generalized letters, each member of the generalized letters was calculated, (i.e., “A” versus “T” and “C” versus “G”), and results were summarized by rules of Table 1. If there was more than one pair of sequences with the minimum distance, the phylogenetic distances were sorted using Kruskal-Wallis statistics in descending order as follows:

(1) $$\begin{array}{c} KW=1-p^{new}-\frac{\sum_{i=1}^{n}\left(1-p_{i}^{original}\right)}{n}, \end{array}$$

where *p* ^new^ was the Kruskal-Wallis test *p* value of new motif profile, *p*_*i*_^original^ was the Kruskal-Wallis test *p* value of *i*th original sequence covered by the new motif, and *n* was the number of original sequences or their antisense complementary sequences covered by the new motif. The generalized motif (and its antisense complementary motif) was composed of a group of original sequences; the original sequences with profiling were defined as covered by the new motifs. Profile of the new motif was calculated as follows:

(2) $$\begin{array}{c} \text{profiling\_motif}=\text{profile}\left(\;\text{:, covered}\;\right)*\text{LDA\_weights}, \end{array}$$

where “profile(:, covered)” were the profiles of original sequences covered by the new motif (i.e., rows were samples and columns were the covered original sequences), and LDA_weights were the linear combination weights calculated by Linear Discriminant Analysis (LDA) [16] method with maximum Fisher's Rayleigh quotient (shown in “Linear Discriminant Analysis” part in the other method). Therefore profiles of the covered sequences were replaced by the profile of new motif.

With the clustering rule, the original sequences could be transformed into the generalized motifs. To suit for high-dimension characteristics of text data, first *m*th nonredundant pairs of sequences were combined to new motifs, where *m* = square  root (Sqrt in short) of the total number of sequences with the same length. The batch computing method could accelerate motif finding part of PMF and avoid overfitting the training data [17]. Therefore the generalized motifs were found iteratively until all sites changed into “N.”

After finding motifs with the same length, different length (e.g., from two to six) motifs needed to be sorted using motif sorting part of PMF by integrating Kruskal-Wallis *p* value [18] and specificity in descending order. For each motif, the specificity was calculated as follows:

(3) $$\begin{array}{c} specificity=\frac{\sum_{i=1}^{K}\left(1-x_{i}/4\right)}{K}, \end{array}$$

where *K* was the length of each sequence and *x*_*i*_ was the number of members of *i*th site's (generalized) letter. Therefore each motif could be sorted in descending order as follows:

(4) $$\begin{array}{c} 1-p\text{ value}+specificity. \end{array}$$

With suitable motifs, original profiles could be merged into profiles of motifs by (2), and covered profiles could be deleted so dimensions reduction would be performed. The more original sequences were replaced by generalized motifs; the linear bias was getting greater, but variance was getting lower, and vice versa. To compromise between the bias and variance criteria, model evaluation part was performed to select necessary motifs. Other than training errors calculated by (5-fold proportional) cross validation, number of dimensions was also considered. Therefore, at most the first *p* (*p* = Sqrt{*N*}) models with minimum training errors were set as candidate models to be evaluated and combined with dimensions (in descending order). The number of dimensions was considered as the logarithm penalty [17], together with the training errors, so the minimum value model was selected as follows:

(5) i⟵arg⁡min⁡training_error+log⁡dimension∗logN∗kN∗k,

(6) N=min⁡number_training_data,b,

(7) k=min⁡dimensionmax⁡dimension,

where *b* was the number of candidate models with lower dimensions than the model minimum training error.

**Figure 1:**
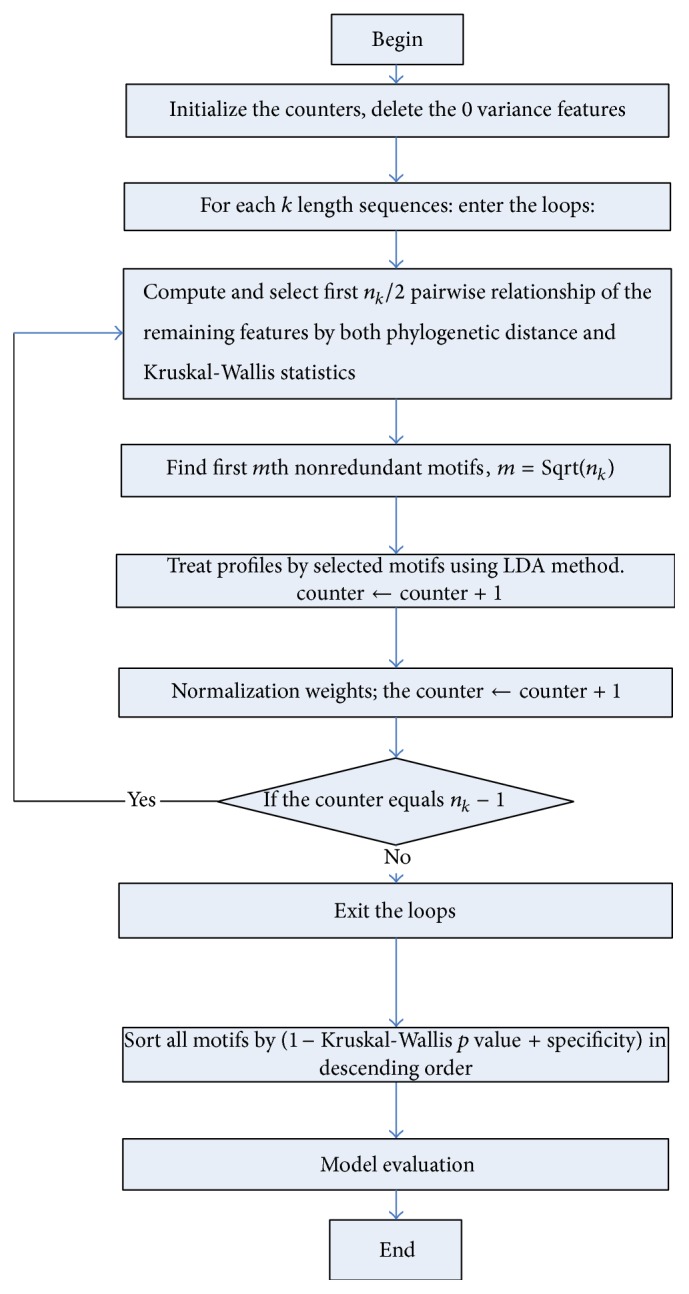
PMF algorithm flowchart.

The pipeline of the proposed algorithm is described in detail below and its flowchart was shown in Figure 1.

**Step 1 (initialization).**

Delete features (sequences) with zeros variance profile. Set counters of 2 to 6 length sequences to zero.

**Step 2 .**

Enter each loop from Step 3 to Step 5 until the counter reaching *n*_*k*_ − 1 for length of sequences is from 2 to 6, respectively (i.e., *n* = 2,3, 4,5, 6).

**Step 3 .**

Select and sort first *kn*/2 pairs of sequences with minimum phylogenetic distance combined with profile Kruskal-Wallis statistics in descending order as (1).

**Step 4 (remove redundancy of the selected pairs).**

For each current sorted pairs, delete any later selected pairs having intersection with the current one. Finally select *m* = Sqrt(*kn*) pairs at most.

**Step 5 .**

Merge the final selected pairs of sequences into the generalized sequences/motifs. Combine profiles of original sequences covered by the generalized motif using Linear Discriminant Analysis (LDA) method with maximum Fisher's Rayleigh quotient value. Counter ← Counter + *m*.

**Step 6 .**

Sort the motifs by the specificity and Kruskal-Wallis *p* value in descending order using (4).

**Step 7 .**

Evaluate models by (5). Treat original profiles by selected suitable motifs using (2).

### 2.3. Other Methods

#### 2.3.1. Kruskal-Wallis Test

Kruskal-Wallis [18] test is a nonparametric method for testing whether samples originate from the same distribution. The test assumes that all samples from the same group have the same continuous distribution, and they are mutually independent. In this study, Kruskal-Wallis *p* value was used to rank features.

### 2.4. Information Gain Method

Information Gain [19] measures the classification ability of each feature with respect to the relevance with the output class, which is defined as Information Gain = *H*(*S*) − *H*(*S*∣*x*):

(8) HS=−∑s∈Spslog2⁡ps,HS ∣ x=−∑x∈Xpx∑s∈Sps ∣ xlog2⁡ps ∣ x,

where *S* and *x* are features. When measuring the mutual relation between the extracted features and the class, Information Gain is also known as mutual information. *k*-mer counting values were discretized using two thresholds' mean ± std. If more than one sequence was with the same Information Gain value, they were sorted by Kruskal-Wallis *p* value.

### 2.5. Chi-Square Statistic

This method uses the Chi-square statistic to discretize numeric attributes and achieves feature selection via discretization [20]. The Chi-square value is defined as

(9) χ2=∑i=1c ∑j=1kAij−Eij2Eij,Eij=MiBjN,

where *c* is the number of intervals, *k* is the number of classes, *A*_*ij*_ is the number of samples in the *i*th interval and the *j*th class, *M*_*i*_ is the number of samples in the *i*th interval, *B*_*j*_ is the number of samples in the *j*th class, and *N* is the total number of samples. *k*-mer counting values were discretized using two thresholds' mean ± std. If more than one sequence was with the same Chi-square statistic, they were sorted by Kruskal-Wallis *p* value.

### 2.6. Linear Discriminant Analysis

Linear Discriminant Analysis (LDA) is a typical variable transformation method to reduce dimensions [16]. The key step of LDA is to maximize the Rayleigh quotient:

(10) $$\begin{array}{c} J\left(\;W\;\right)=\frac{\alpha^{T}S_{B}\alpha }{\alpha^{T}S_{W}\alpha }, \end{array}$$

where the “between-class scatter matrix” is defined as

(11) $$\begin{array}{c} S_{B}=\underset{k}{\sum }\left(\;p_{k}-1\;\right)\sum \frac{\left(m_{k}-m\right){\left(m_{k}-m\right)}^{\prime }}{\left(K-1\right)} \end{array}$$

and the “within-class scatter matrix” is defined as

(12) $$\begin{array}{c} S_{W}=\overset{K}{\underset{k}{\sum }}\frac{\left(y-m_{k}\right){\left(y-m_{k}\right)}^{\prime }}{\left(N-K\right)}. \end{array}$$

*K* is the number of classes, *p*_*k*_ is the number of the samples within the *k*th class, *m*_*k*_ is the mean value of the sample within the *k*th class, and *m* is the mean value of all the samples.

Traditional LDA requires the total scatter matrix to be nonsingular. To deal with the singularity problems, classical LDA method was modified in a way that a unit diagonal matrix with small weights was added to the within-class scatter matrix, if the scatter matrix is singular [14].

## 3. Results and Discussion

We first performed the PMF algorithm on pneumonia samples. We considered both 2-class problem (pneumonia: CAP + HAP, versus normal) and 3-class problem (HAP, CAP, versus normal). Conventionally, due to the data imbalance, the accuracy for each class was used to measure the classification, which was equivalent to combining the specificity and sensitivity in general classifications. Two widely applied methods, nearest neighbor algorithm (NNA) and support vector machine (SVM), were used to select the optimal classifier set of motifs extracted by PMF for pneumonia samples. Since SVM mainly suits pairwise classifications, normal samples must be discriminated against the pneumonia samples (CAP and HAP) before CAP and HAP were classified in a 3-class problem. To evaluate the performance of our (*k*-mer) motif-oriented method, we compared our results with those of previous methods, including the Feature Merging and Selection algorithm (FMS) based on sequence-microbe associations [14], as well as other *k*-mer counting feature selection algorithms, for example, the Information Gain method [19], Chi-square statistic method [20], and primitive Kruskal-Wallis statistic method [18].

**Figure 2:**
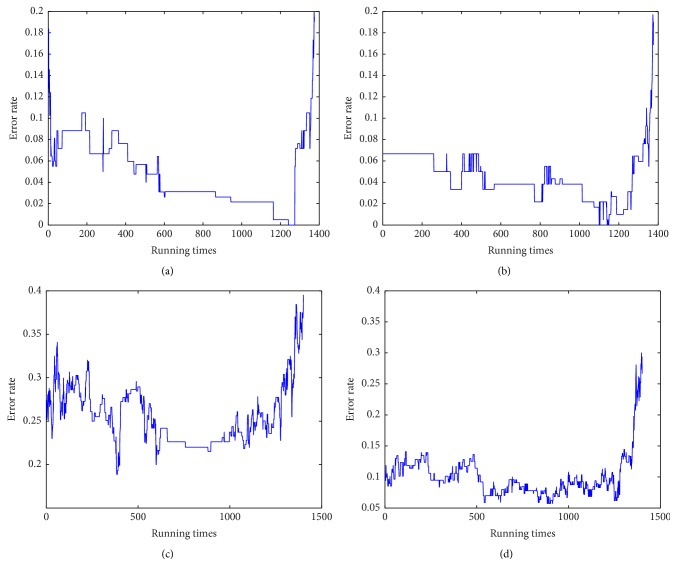
Learning curves of PMF algorithm for 3-class problem with NNA (a), for 3-class problem with SVM (b), for 2-class problem with NNA (c), and for 2-class problem with SVM (d).

Figure 2 showed the learning curves for the training data; combined with logarithm penalty evaluation, the best evaluated models were selected with 1321 and 1369 runs of PMF for the 3-class problem, with SVM and NNA classifiers, respectively. Optimal models for the 2-class problem were selected with 1218 and 1136 runs of PMF (with SVM and NNA). As shown in Tables 2 and 3, our method had the lowest mean error in both 3-class and 2-class problem (with either SVM or NNA combined), compared with the previous methods mentioned earlier.

**Figure 3:**
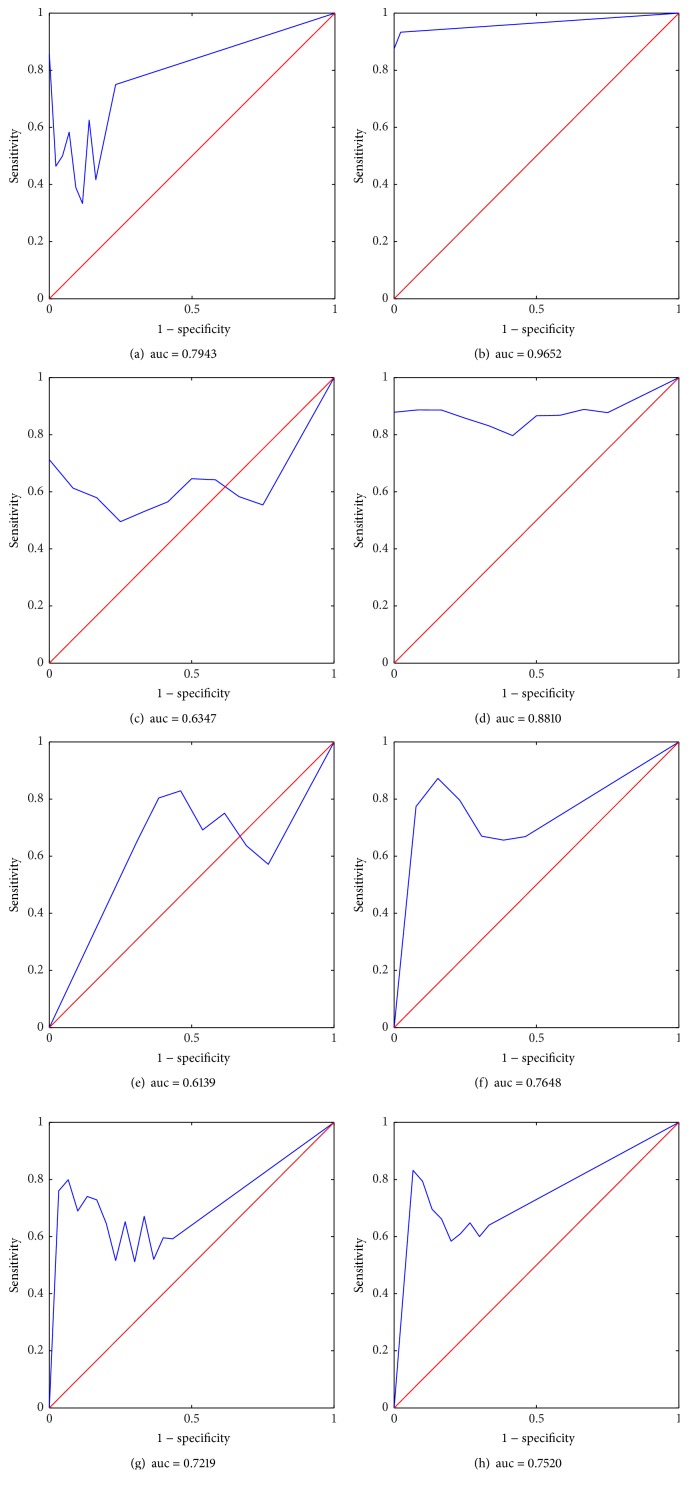
ROC curves of motif finding step of PMF algorithm for pneumonia samples (CAP + HAP) in 2-class problem (a, b), normal samples (c, d), CAP samples in 3-class problem (e, f), and HAP samples in 3-class problem (g, h), with NNA or SVM.

In statistics, a receiver operating characteristic (ROC) curve is the summary of both sensitivity and specificity for various thresholds. ROC was constructed for each subset of features (Figure 3). As shown, the optimal features that are selected under the combined criteria of cross validation and model evaluation possessed high specificity (~80%) with high sensitivity (~70%) for the 3-class problem, indicating the ability of our method. Moreover, even higher specificity and sensitivity were obtained (>0.95) for the 2-class problem. Noteworthy, PMF combined with SVM performed better in the classification; therefore the results derived by PMF with SVM for the *k*-mer counting profiles of pneumonia samples were used for further analysis.

**Figure 4:**
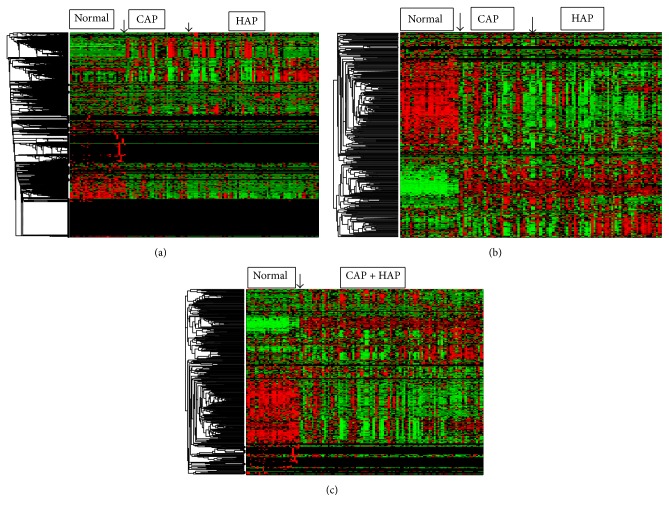
Heat map of *k*-mer counting profiles of original pneumonia data for 3-class problem (a), data after treating by PMF for 3-class problem, (b) and data after treating by PMF for 2-class problem (c). Rows are retained motifs and columns are disease classes. From left to right are 30 normal, 32 CAP, and 71 HAP samples for 3-class problem and 30 normal 103 pneumonia samples for 2-class problem.

Heat map is a frequently used matrix of pairwise sample correlations indicating anticorrelation or correlation using a color scale, that is, green to red.  Figure 4(a) showed that the original data profile was almost invisible for patterns or sample classifications, after being analyzed by our method, since the original feature space had been reduced to a much smaller space spanned by a few features (with the most important variances retained). Therefore as shown in Figures 4(b) and 4(c), the heat maps of the samples were much clearer with high resolutions for classifications.

**Figure 5:**
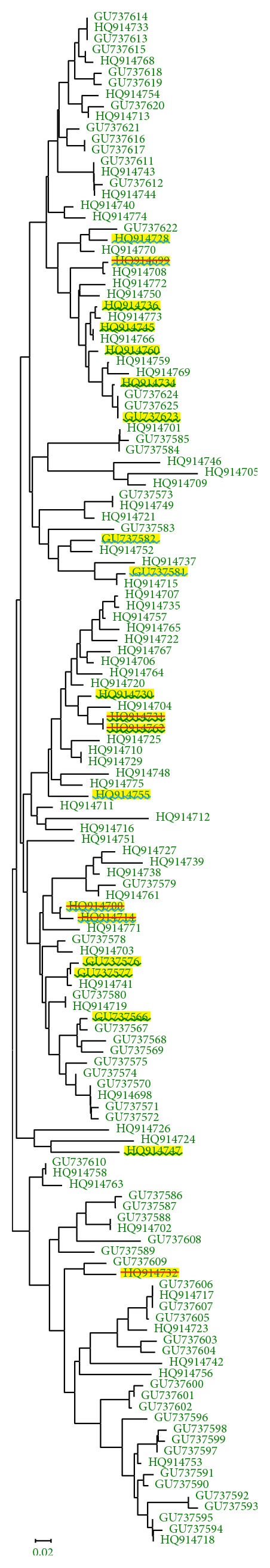
Phylogenetic relationship of identified microbiota signatures. Identified microbes matched by significant motifs are highlighted with underline (“KCTCWT”) or strikethrough (“WTCGTC”).

Profiles with reduced dimensions obtained by PMF were sorted according to the Kruskal-Wallis *p* values. The top 5 motifs with *p* value < 0.05 in 3-class problem were “KCTCWT,” “TTCGHT,” “CGATCS,” “TCWCTA,” and “TTWCGC”. Sequences (including antisense complementary sequences) covered by the first motif “KCTCWT” (*p* value = 0.0126) were matched to the microbe taxonomic results of Zhou et al. [2]. 6 out of 19 matched microbes were among the top 20 genera suspiciously contributing to pneumonia [2] (Table S1). 10 out of the remaining 13 microbes were also related to pneumonia [21–30] (Table S1). The 2 motifs with *p* value < 0.05 in 2-class problem were “WTCGTC” and “ATCWCT”. Sequences covered by first one “WTCGTC” (*p* value = 0.0288) were matched to the published taxonomic data [2]. Five out of 6 matched microbes were related to pneumonia [2, 29, 30] (Table S2). Furthermore, by pinpointing the 25 (e.g., 19 + 6) matched microbes in a phylogenetic tree constructed from the published taxonomic data (using MEGA6 software [31] with minimum distance method), we observed that distribution of the microbes was dispersed, indicating the diverse functions performed by microbiota for human (Figure 5).

Our method was also tested on *k*-mer counting profiles from dental decay sample. These samples were collected from saliva and dental plaques separately. Combined with logarithm penalty evaluation, the best evaluated models were selected with 2330 and 2485 runs of PMF for dental plaques (with SVM) and saliva samples (with NNA), respectively (Figure  S1). The results showed that our method could also select suitable classifiers and perform better on the test data than the previous and other methods (Tables S3 and S4).

## 4. Conclusions

In this paper, we presented the PMF method to analyze the align-free *k*-mer counting profiles of 16S rRNA microbial data. The improved pipeline systematically analyzed relevance between each pair of sequences using minimum distance phylogenetic trees. Moreover, PMF also considered relationships between *k*-mer counting profiles and the disease status. In addition, by combining original profiles using the LDA method, PMF learned profiles of text strings suitable for disease classification. Batching method also accelerated PMF and avoided overfitting of training data. As a result, via combing characteristics of sequences and classification statistics of text profiles, PMF selected suitable motifs to evaluate metagenome characteristics of human microbiota disease.

In conclusion, we developed an improved motif-based text mining algorithm, and the new pipeline was verified by both pneumonia and dentes cariosus samples. As the classification results have shown, it was demonstrated that PMF was an effective approach for finding informative motifs from training data, and it was validated well compared with the previous study and other widely used methods. PMF performed well and it could extend evolutionary/phylogenetic approaches to perform supervised learning on microbiota text data to discriminate disease/pathology status.

## Supplementary Material

File S1 and S2: *k*-mer counting results of pneumonia and dental caries data. In File S1, 0 indicates normal sample, 1 indicates CAP and 2 indicates HAP in the last raw. In File S2, 1 indicates dental plaques patient, 2 indicates dental decay control sample, 3 indicates saliva patient, and 4 indicates saliva control sample in the last raw.
File S3: Other Supplementary Materials. Figure S1 Learning curves of motif finding step of PMF algorithm for dental plaques samples with SVM (a) and saliva samples with NNA (b). Table S1: Microbiota signatures (“KCTCWT”) in 3-class problem in pneumonia samples. Table S2: Microbiota signatures (“WTCGTC”) in 2-class problem in pneumonia samples. Table S3: Classification results of dental decay data from decay plaques samples. Table S4: Classification results of dental decay data from saliva samples.

## Figures and Tables

**Table 1:** Alphabet of generalized letters.

Letter	Members	Antisense complementary letter
R	AG	Y
Y	CT	R
W	AT	W
M	AC	K
K	GT	M
S	CG	S
H	ACT	D
B	CGT	V
V	ACG	B
D	CGT	H
N	ACGT	N

**Table 2:** Classification results of pneumonia data in 3-class problem.

Method	Error rate		Dimension	Feature
	On training data	On test data		
SVM/FMS	0.1895	0.2637	29	Microbes
SVM/PMF	0.062	0.0756	411	Sequences
SVM/Kruskal-Wallis	0.1187	0.5273	272	Sequences
SVM/Information Gain	0.143	0.2124	12	Sequences
SVM/Chi-square statistic	0.1743	0.5909	1280	Sequences
SVM	0.2187	0.3812	4390	Sequences
NNA/FMS	0.2013	0.3406	112	Microbes
NNA/PMF	0.2152	0.2081	786	Sequences
NNA/Kruskal-Wallis	0.2718	0.3363	85	Sequences
NNA/Information Gain	0.2354	0.4141	39	Sequences
NNA/Chi-square statistic	0.2649	0.3107	69	Sequences
NNA	0.442	0.6162	4390	Sequences

**Table 3:** Classification results of pneumonia data in 2-class problem.

Method	Error rate		Dimension	Feature
	On training data	On test data		
SVM/FMS	0.0922	0.1279	42	Microbes
SVM/PMF	0	0	551	Sequences
SVM/Kruskal-Wallis	0.01	0	28	Sequences
SVM/Information Gain	0	0.0116	26	Sequences
SVM/Chi-square statistic	0.01	0.0417	127	Sequences
SVM	0.0667	0.0116	4390	Sequences
NNA/FMS	0.1279	0.2393	20	Microbes
NNA/PMF	0	0.0833	361	Sequences
NNA/Kruskal-Wallis	0.0167	0.125	13	Sequences
NNA/Information Gain	0.0214	0.125	12	Sequences
NNA/Chi-square statistic	0.0381	0.125	26	Sequences
NNA	0.2667	0.5	4390	Sequences

## Abbreviations

PMF: Phylogenetic Tree-Based Motif Finding algorithm
FMS: Feature Merging and Selection algorithm
LDA: Linear Discriminant Analysis
HAP: Hospital-acquired pneumonia
CAP: Community-acquired pneumonia
NNA: Nearest neighbor algorithm
SVM: Support vector machine.
