# Supplementary material for: Motif-Based Text Mining of Microbial Metagenome Redundancy Profiling Data for Disease Classification
Source: Biomed Res Int. 2016 Feb 14;2016:6598307. doi: 10.1155/2016/6598307 (PMC4769744; doi:10.1155/2016/6598307)
Supplement: Supplementary file 1 — File S1 and S2: k-mer counting results of pneumonia and dental caries data. In File S1, 0 indicates normal sample, 1 indicates CAP and 2 indicates HAP in the last raw. In File S2, 1 indicates dental plaques patient, 2 indicates dental decay control sample, 3 indicates saliva patient, and 4 indicates saliva control sample in the last raw. File S3: Other Supplementary Materials. Figure S1 Learning curves of motif finding step of PMF algorithm for dental plaques samples with SVM (a) and saliva samples with NNA (b). Table S1: Microbiota signatures (“KCTCWT”) in 3-class problem in pneumonia samples. Table S2: Microbiota signatures (“WTCGTC”) in 2-class problem in pneumonia samples. Table S3: Classification results of dental decay data from decay plaques samples. Table S4: Classification results of dental decay data from saliva samples. [file 6598307.f1.zip › supplemental file/supplemental material.pdf]

Figure S1 Learning curves of motif finding step of PMF algorithm for dental plaques samples with SVM (a) and saliva samples with NNA (b)

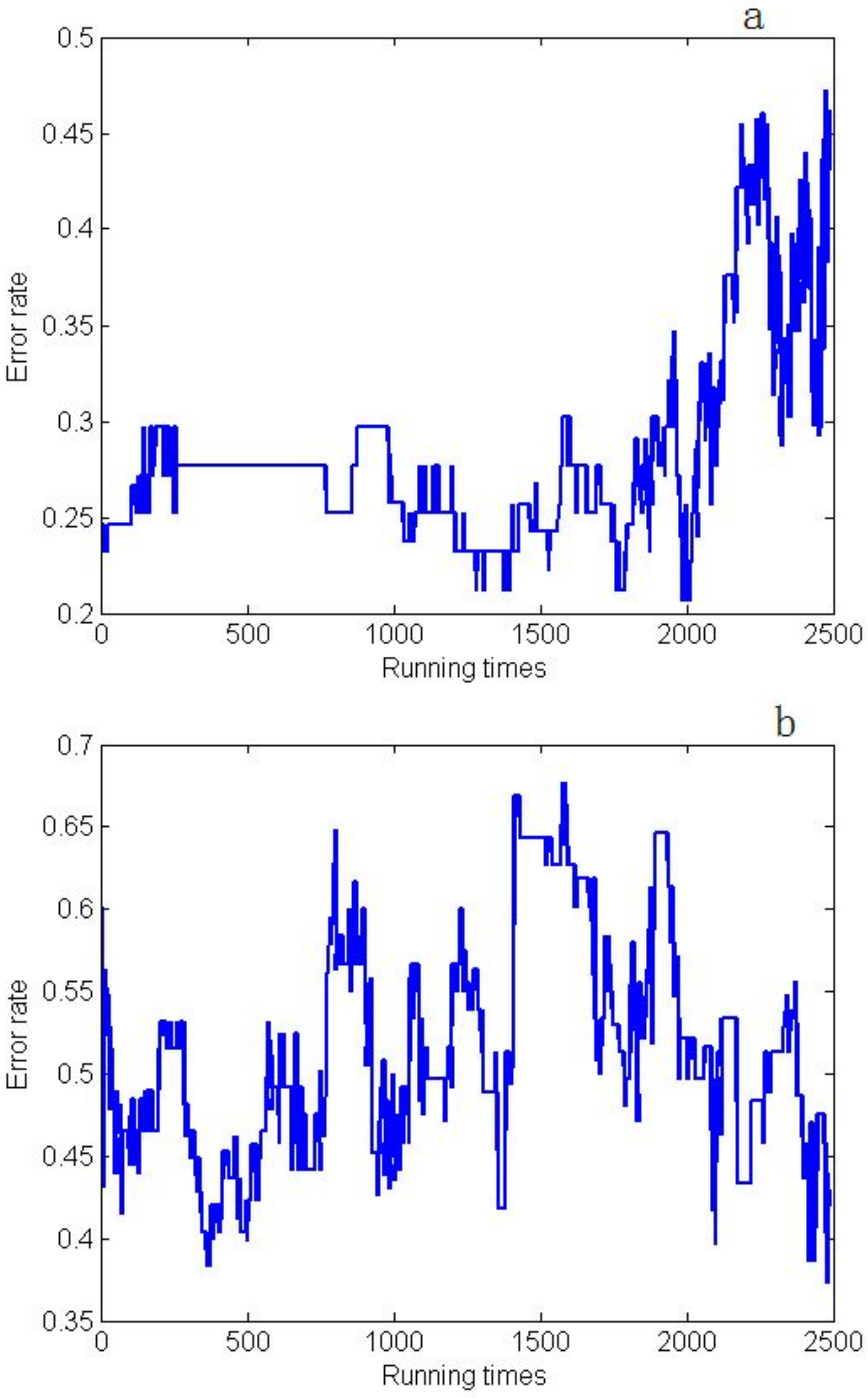

Table S1: Microbiota signatures ('KCTCWT') in 3-class problem in pneumonia samples

Name	Id [2]	Relate to pneumonia
Actinomyces	GU737566	*
Pseudonocardia	GU737576	[21]
Mycobacterium	GU737577	*
Parvimonas	GU737581	*
Eubacterium	GU737582	[22]
Acinetobacter	GU737623	*
Aggregatibacter	HQ914699	*
Atopobium	HQ914700	*
Olsenella	HQ914714	[23]
Amaricoccus	HQ914728	
Anoxybacillus	HQ914730	[24]
Brevibacillus	HQ914731	
Enhydrobacter	HQ914734	[25]
Ferrimonas	HQ914736	
Serratia	HQ914745	[26]
Thermus	HQ914747	[27]
Peptococcus	HQ914755	[28]
Aeromonas	HQ914760	[29]
Brevundimona	HQ914762	[30]

* top 20 genera suspiciously contributing to pneumonia [2]

Table S2: Microbiota signatures (‘WTCGTC') in 2-class problem in pneumonia samples

Name	Id [2]	Relate to pneumonia
Aggregatibacter	HQ914699	*
Atopobium	HQ914700	*
Olsenella	HQ914714	*
Brevibacillus	HQ914731	
Chryseobacterium	HQ914732	[30]
Brevundimonas	HQ914762	[29]

* top 20 genera suspiciously contributing to pneumonia [2]

Table S3: Classification results of dental decay data from decay plaques samples

Method	Error rate		Dimension
	On training data	On test data	
SVM/FMS	0.3385	0.4444	13
SVM/PMF	0.2876	0.2308	402
SVM/KruskalWallis	0.2319	0. 3654	2705
SVM/InformationGain	0.2121	0.5192	132
SVM/Chi-square statistic	0.2462	0.3654	2997

Table S4: Classification results of dental decay data from saliva samples

Method	Error rate		Dimension
	On training	On test data	

	data		
SVM/FMS	0.2583	0.4118	46
NNA/PMF	0.42	0.1169	40
NNA/KruskalWallis	0.3617	0. 3766	97
SVM/InformationGain	0.38	0.4221	2705
SVM/Chi-square statistic	0.2567	0.3961	122
